# Matrix stiffness regulates migration of human lung fibroblasts

**DOI:** 10.14814/phy2.13281

**Published:** 2017-05-14

**Authors:** Shuichi Asano, Satoru Ito, Kota Takahashi, Kishio Furuya, Masashi Kondo, Masahiro Sokabe, Yoshinori Hasegawa

**Affiliations:** ^1^ Department of Respiratory MedicineNagoya University Graduate School of MedicineNagoyaJapan; ^2^ Mechanobiology LaboratoryNagoya University Graduate School of MedicineNagoyaJapan; ^3^ Department of Respiratory Medicine and AllergologyAichi Medical UniversityNagakuteJapan

**Keywords:** Matrix stiffness, mechanotransduction, migration, pulmonary fibrosis, *α*‐smooth muscle actin

## Abstract

In patients with pulmonary diseases such as idiopathic pulmonary fibrosis and severe acute respiratory distress syndrome, progressive pulmonary fibrosis is caused by dysregulated wound healing via activation of fibroblasts after lung inflammation or severe damage. Migration of fibroblasts toward the fibrotic lesions plays an important role in pulmonary fibrosis. Fibrotic tissue in the lung is much stiffer than normal lung tissue. Emerging evidence supports the hypothesis that the stiffness of the matrix is not only a consequence of fibrosis, but also can induce fibroblast activation. Nevertheless, the effects of substrate rigidity on migration of lung fibroblasts have not been fully elucidated. We evaluated the effects of substrate stiffness on the morphology, *α*‐smooth muscle actin (*α*‐SMA) expression, and cell migration of primary human lung fibroblasts by using polyacrylamide hydrogels with stiffnesses ranging from 1 to 50 kPa. Cell motility was assessed by platelet‐derived growth factor (PDGF)‐induced chemotaxis and random walk migration assays. As the stiffness of substrates increased, fibroblasts became spindle‐shaped and spread. Expression of *α*‐SMA proteins was higher on the stiffer substrates (25 kPa gel and plastic dishes) than on the soft 2 kPa gel. Both PDGF‐induced chemotaxis and random walk migration of fibroblasts precultured on stiff substrates (25 kPa gel and plastic dishes) were significantly higher than those of cells precultured on 2 kPa gel. Transfection of the fibroblasts with short interfering RNA for *α*‐SMA inhibited cell migration. These findings suggest that fibroblast activation induced by a stiff matrix is involved in mechanisms of the pathophysiology of pulmonary fibrosis.

## Introduction

Irreversible or progressive pulmonary fibrosis is a pathological feature of dysregulated wound healing after lung inflammation or damage in patients with idiopathic pulmonary fibrosis (IPF), radiation pneumonitis, and severe acute respiratory distress syndrome (ARDS). Pulmonary fibrosis is characterized by excessive deposition of extracellular matrix (ECM) and accumulation of lung fibroblasts via upregulation of proliferation and migration. The neoexpression of *α*‐smooth muscle actin (*α*‐SMA) is a distinctive marker of the activated fibroblasts called myofibroblasts, which results in the contraction and possibly stiffening of collagenous ECM (Kuhn and McDonald 1991; Flaherty et al. 2003; White et al. 2003a; Hinz 2012).

In addition to well‐established mediators, specifically transforming growth factor‐*β* (TGF‐*β*), mechanical cues such as contractile force, shear stress, compression, stretch, and matrix stiffness are involved in the mechanisms underlying the pathogenesis of fibrosis (Booth et al. 2012; Hinz 2012; Duscher et al. 2014). The elastic modulus of the fibrotic lung (15–100 kPa) is much stiffer than that of normal lung parenchyma (0.5–5 kPa) (Liu et al. 2010; Booth et al. 2012; Hinz 2012; Southern et al. 2016). In general, cells are able to sense and respond to the stiffness of the substrates on which they are growing (Kobayashi and Sokabe 2010). It has been demonstrated that motility, spreading, proliferation, and differentiation of cells are critically influenced by substrate rigidity (Lo et al. 2000; Engler et al. 2006; Liu et al. 2010; Grinnell and Ho 2013). In lung fibroblasts, stiff substrates corresponding to pathologically fibrotic tissues induce differentiation to a highly synthetic and contractile phenotype (Huang et al. 2012; Marinkovic et al. 2012; Zhou et al. 2013). The motility of fibroblasts toward fibrotic lesions plays an important role in tissue fibrosis (Tschumperlin 2013). However, the effects of substrate stiffness on motility of lung fibroblasts have not been fully elucidated.

This study was designed to determine the effects of substrate stiffness on the regulation of cellular properties of primary human lung fibroblasts. We postulated that when cultured on stiff substrates, fibroblasts gain motility by upregulating expression of *α*‐SMA. To this end, we used polyacrylamide gels with different stiffnesses as substrates (Yeung et al. 2005; Tse and Engler 2010).

## Materials and Methods

### Cells

Primary cultures of normal human lung fibroblasts from three different donors were obtained from Lonza (Walkersville, MD). The cells were cultured on plastic dishes (Nunc™ Cell Culture/Petri Dishes, Thermo Fisher Scientific, Waltham, MA), and maintained in culture medium (FGM‐2 BulletKit; Lonza) in an atmosphere of 5% CO_2_ and 95% air at 37°C in accordance with the manufacturer's procedures (Murata et al. 2014). Cells of passages 4–6 were used.

### Polyacrylamide hydrogels

Commercially available polyacrylamide hydrogels bound to six‐well polystyrene plates or polystyrene dishes of various stiffnesses (1, 2, 8, 25, or 50 kPa) coated with type I collagen were used (Softwell; Matrigen Life Technologies, Brea, CA).

### Quantitative analysis of cell morphology and numbers

Fibroblasts were cultured on polyacrylamide gels of different stiffnesses for 4 to 72 h. FGM‐2 cell culture medium was replaced at 4, 16, and 72 h after seeding, then phase‐contrast images of 10 randomly chosen fields of view per condition per experiment were taken using an inverted microscope (CKX41; Olympus, Tokyo, Japan). Cell projection area, perimeter, aspect ratio, and circularity were determined from manual tracings of individual cells using NIH ImageJ v1.33. The aspect ratio was calculated as the major axis/minor axis ratio. Circularity was calculated as (eq. 1):

(1) $$\text{Circularity}=4\pi \times \frac{\text{Cell area}}{{\text{perimeter}}^{2}}$$

Fifty cells from five independent experiments were analyzed for each substrate. Cell number was counted manually.

### Cell viability assay

Cell viability was assessed by a Live/Dead Viability/Cytotoxicity Assay Kit (L3224; Thermo Fisher Scientific). Briefly, the live cells were stained with fluorescent calcein and dead cells with ethidium bromide (EthD‐1) for 15 min at 37°C, and then were visualized using a fluorescence microscope (IX83; Olympus). Approximately 100 cells were manually counted for the assay.

### Immunofluorescence staining

Fibroblasts grown on polyacrylamide gels or plastic dishes (Thermo Fisher Scientific) were fixed and permeabilized for 30 min with 4% formaldehyde and 0.2% Triton X‐100 in cytoskeleton‐stabilizing buffer (137 mmol/L NaCl, 5 mmol/L KCl, 1.1 mmol/L Na_2_HPO_4_, 0.4 mmol/L KH_2_PO_4_, 4 mmol/L NaHCO_3_, 2 mmol/L MgCl_2_, 5.5 mmol/L glucose, 2 mmol/L EGTA, and 5 mmol/L PIPES; pH 6.1). This was followed by blocking with 1% bovine serum albumin (BSA) in cytoskeleton‐stabilizing buffer for 60 min (Morioka et al. 2011; Hirata et al. 2015). Then, the cells were incubated with a mouse polyclonal anti‐*α*‐SMA antibody (dilution 1:400, a2547; Sigma‐Aldrich, St. Louis, MO) in a cytoskeleton‐stabilizing buffer containing 1% BSA for 40 min, washed, and further incubated with a goat anti‐mouse secondary antibody (dilution 1:1000, A‐11001; Thermo Fisher Scientific) for 40 min at room temperature. Filamentous actin (F‐actin) and nuclei were stained with rhodamine–phalloidin (dilution 1:1000, R415; Thermo Fisher Scientific) and 4,6‐diamino‐2‐phenylindole (DAPI) (dilution 1:1000, D523; Dojin, Kumamoto, Japan) for 40 min at room temperature. The immunofluorescence images were obtained using an upright laser scanning confocal microscope (A1RMP; Nikon, Tokyo, Japan), with a ×25/1.2 NA Plan Apo violet‐corrected water immersion objective.

### Western blotting

Protein concentrations of cellular lysates were measured by using a Bio‐Rad protein assay reagent kit (Bio‐Rad, Hercules, CA). Equal amounts of lysates, adjusted for protein concentration, were resolved by SDS‐PAGE using a 5–20% linear gradient running gel (Wako, Osaka, Japan). Proteins were transferred to nitrocellulose membranes, and membranes were blocked in 5% skim milk for 1 h at room temperature. Immunoblotting was performed using antibodies against *α*‐SMA (dilution 1:400, ab5694; Abcam, Tokyo, Japan), phospho‐myosin light chain (p‐MLC) (dilution 1:1000, M6068; Sigma‐Aldrich), phospho‐focal adhesion kinase (p‐FAK) (dilution 1:1000, 8566p; Cell Signaling Technology, Danvers, MA), and GAPDH (dilution 1:1000, 8884s; Cell Signaling Technology). Immunodetection was accomplished using a HRP‐conjugated donkey anti‐rabbit secondary antibody (dilution 1:2000, NA934; GE Healthcare, Buckinghamshire, UK) or sheep anti‐mouse secondary antibody (dilution 1:2000, NA931, GE Healthcare), and an Enhanced Chemiluminescence kit (dilution 1:5000, Amersham Biosciences, Piscataway, NJ). The intensity was quantified by using Quantity One software ver. 4.6.9. (Bio‐Rad).

### Transwell migration assay

A modified Boyden chamber (8‐*μ*m pore filter, 24‐well cell clusters) (Chemotaxicell; Kurabo, Osaka, Japan) coated with type I collagen (Nitta Gelatin, Inc., Osaka, Japan) were used for the chemotaxis assay (Suganuma et al. 2012; Aso et al. 2013). Fibroblasts were cultured on polyacrylamide gels or plastic dishes coated with 10 *μ*g/mL type I collagen in DMEM/F‐12 cell culture medium (Thermo Fisher Scientific) containing 10% FBS for 4 days. The cells were brought to a quiescent state overnight by incubation in DMEM/F‐12 containing 0.1% FBS. The cells were trypsinized, suspended in 400 *μ*L DMEM/F‐12 containing 0.1% FBS (2 × 10^5^ cells/mL), and placed in the upper chamber. PDGF‐BB (P3201; Sigma‐Aldrich) prepared in 0.1% BSA in phosphate‐buffered saline (PBS) dissolved in DMEM/F‐12 containing 0.1% FBS was inserted in the wells of the lower chamber. After incubation with PDGF‐BB (10 ng/ mL) for 6 h at 37°C, the nonmigrated cells on the upper surface of the filter were scraped off with a cotton‐tipped applicator. The migrated cells were fixed and stained with Diff‐Quik (Sysmex, Kobe, Japan) and mounted on glass slides. Cells in five fields per chamber were counted using an inverted microscope (CKX41; Olympus) with a 10× objective. In the Results section, “*n*” refers to the number of experiments performed. Each experimental condition was tested in duplicate. Solvents did not affect cell migration at the concentrations used (0.1%/vol.).

### Random walk cell migration assay

Random walk cell migration was assessed using an imaging system (Liu et al. 2010; Southern et al. 2016). Briefly, images were obtained using a time‐lapse microscopy system (IncuCyte ZOOM; Essen BioScience, Ann Arbor, MI), consisting of a CO_2_ incubator and an inverted microscope with a 10× objective. Fibroblasts were cultured on polyacrylamide gels or plastic dishes coated with type I collagen in DMEM/F‐12 cell culture medium containing 10% FBS for 4 days. Cells were trypsinized and seeded onto plastic dishes coated with type I collagen for 1 h at a density of 1000 cells/cm^2^ to reduce cell–cell contacts. Cell viability and adherent cell numbers after transfer to the dishes were not different between conditions. Then, phase‐contrast images of single cells were captured at 20‐min intervals over a 12‐h period. Displacements of cells and xy centroids were determined using MetaMorph 6.1 cell‐tracking module (Molecular Devices, Tokyo, Japan). Total distance migrated and distance from the start point were assessed. Cells that came into contact with each other or divided were excluded from the analysis as described in previous reports by other laboratories (Harms et al. 2005; Liu et al. 2010).

### Transfection with siRNA

RNA interference was performed using short interfering RNAs (siRNAs) specific for the *α*‐SMA gene (*ACTA2*) (HSS100114; Thermo Fisher Scientific) and scrambled siRNA (#12935300; Thermo Fisher Scientific). Transfection reagent–siRNA complexes were prepared by using Lipofectamine RNAiMAX regent (#13778150; Thermo Fisher Scientific). The cells seeded on collagen‐coated plastic dishes were transfected with siRNA sequences for 10 nmol/L *α*‐SMA (siSMA) or scrambled siRNA in culture medium without antibiotics. To avoid the effects of cell adhesion, the transfection was performed at 4 h after seeding. To minimize the possibility of off‐target effects, three different siRNAs targeting *α*‐SMA were tested. Extraction of cell lysate for Western blotting and cell migration assays were performed 72 h after siRNA transfection (Suganuma et al. 2012; Takahara et al. 2014).

### Statistical analysis

Data are expressed as means ± SD. Unpaired *t*‐test or analysis of variance (ANOVA) followed by Bonferroni's or Games‐Howell's post hoc tests were used to evaluate the statistical significance. *P *<* *0.05 was considered statistically significant. Statistical analyses were performed using SPSS ver. 24 (SPSS, Inc., Chicago, IL).

## Results

### Effects of substrate stiffness on cell morphology

**Figure 1:**
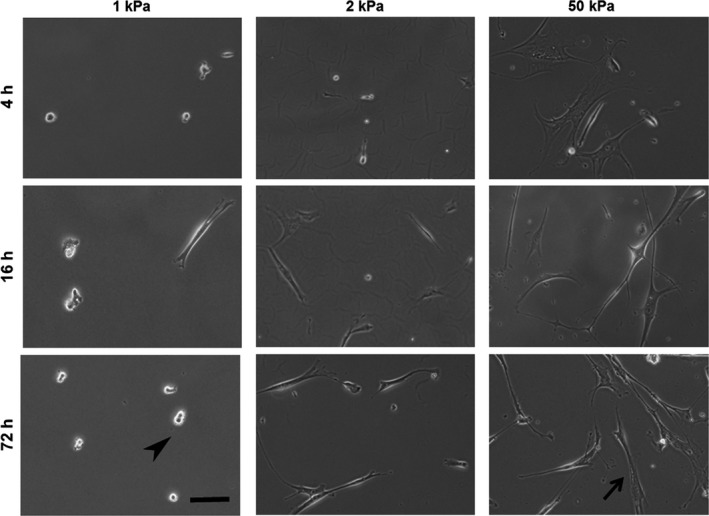
Time‐dependent changes of cell morphology on different substrates. Representative phase‐contrast images of normal human lung fibroblasts cultured on polyacrylamide hydrogels of different stiffnesses (1, 2, and 50 kPa) for 4, 16, and 72 h. Cell growth and spreading were suppressed on the softest (1 kPa) gels. Arrowhead and arrow show nonspreading and spreading cells, respectively. Bar = 100 *μ*m.

**Figure 2:**
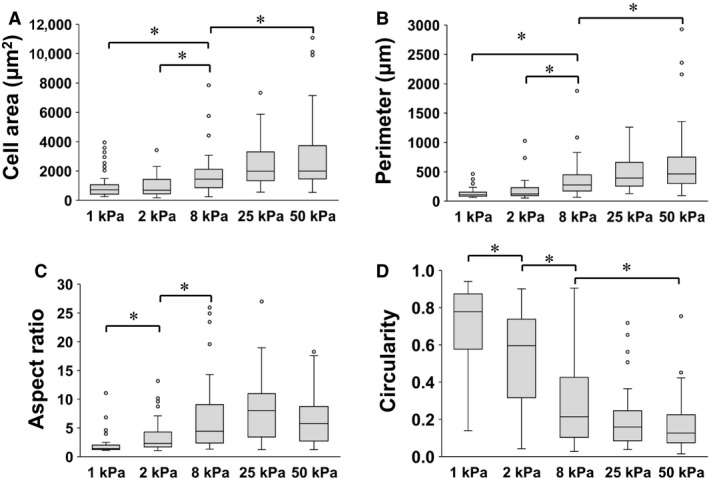
Effects of substrate stiffness on morphology of fibroblasts. Fibroblasts were cultured on polyacrylamide hydrogels of different stiffnesses (1, 2, 8, 25, and 50 kPa) for 16 h, and 50 cells each from three independent experiments were evaluated using ImageJ software. Cell area (A), perimeter (B), aspect ratio (C), and circularity (D) of the cells cultured on different stiffnesses (1, 2, 8, 25, and 50 kPa) of polyacrylamide hydrogels are compared. Boxes represent the 25th and 75th percentiles; whiskers indicate 10th and 90th percentiles. *Significantly different between the groups (P < 0.05).

Different stiffnesses of polyacrylamide gels ranging from 1 to 50 kPa that mimic the physical properties of normal (1 and 2 kPa) and fibrotic (8, 25, and 50 kPa) lung microenvironments (Liu et al. 2010; Liu and Tschumperlin 2011) were used. Figure 1 shows representative morphological images of human lung fibroblasts cultured on collagen I‐coated polyacrylamide gels of different stiffnesses (1, 2, and 50 kPa) for 4, 16, and 72 h. Fibroblasts did not elongate on 1 kPa gel. Cells on 2 kPa gels exhibited mostly round shapes at 4 h, but several cells became spindle‐shaped at 72 h. Cells on 50 kPa polyacrylamide gel spread with dendritic extensions at 4 h. Next, effects of substrate stiffness on morphological changes were quantified. Cell projection area, perimeter, aspect ratio, and circularity were measured 16 h after seeding on the substrates (1–50 kPa) (Fig. 2). Cell projection area, perimeter, and aspect ratio increased and circularity decreased in a stiffness‐dependent manner.

**Figure 3:**
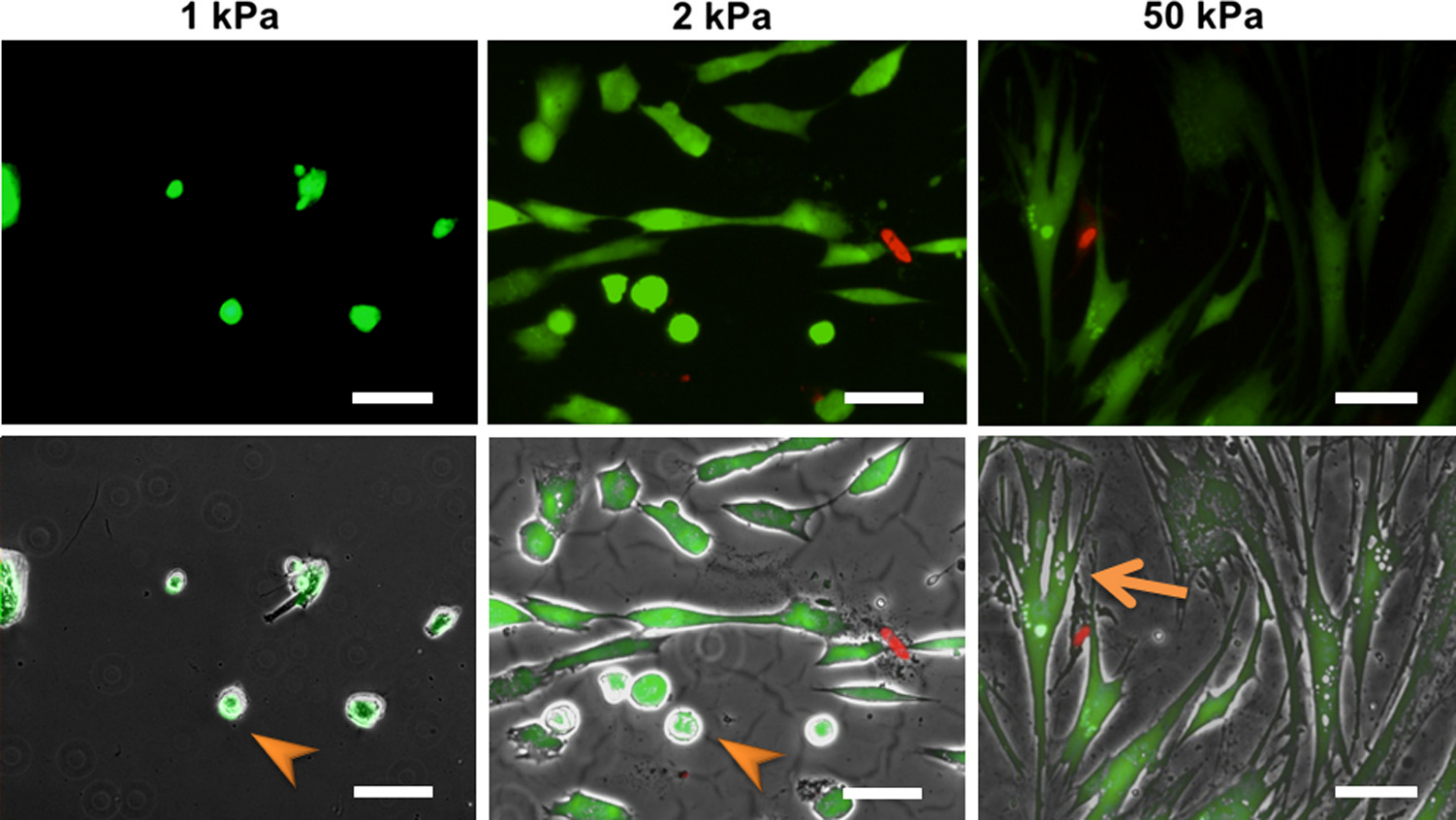
Effects of substrate stiffness on cell viability. *Upper images*: Representative fluorescent images of fibroblasts cultured on 1 (left), 2 (middle), and 50 kPa (right) polyacrylamide hydrogels for 2 days. *Lower images*: Merged images of the fluorescent and phase‐contrast images. Live cells were stained with fluorescent calcein (green) and dead cells with ethidium homodimer‐1 (red). Nonspreading cells on the 1 and 2 kPa gels (arrows in left and middle images) were still alive. The arrowhead (right image) indicates a spread cell. Bar = 100 *μ*m.

Next, cell viability was assessed by fluorescent calcein and EthD‐1 staining 48 h after fibroblasts were seeded on 1, 2, and 50 kPa gels. Both elongated and round cells on 1, 2, and 50 kPa gels were viable (Fig. 3). Ratios of live cells stained with fluorescent calcein to total cells on 1, 2, and 50 kPa gels were 92.7%, 91.3%, and 94.8%, respectively.

### Effects of substrate stiffness on cell proliferation

**Figure 4:**
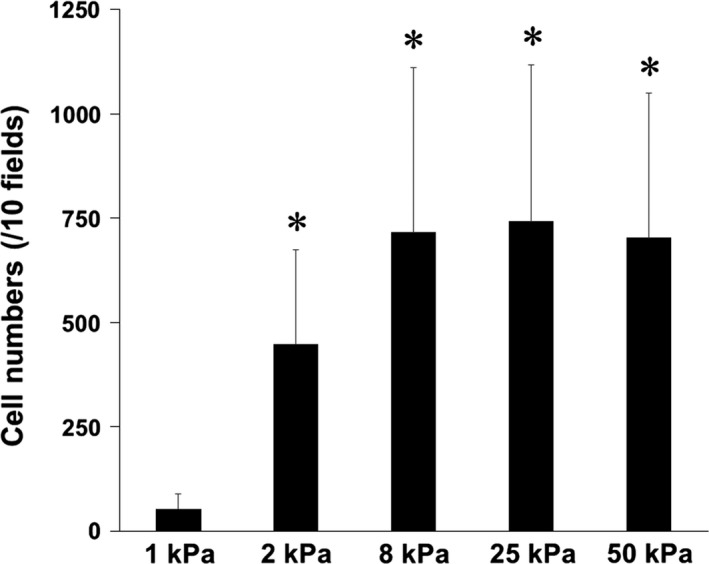
Effects of substrate stiffness on cell proliferation. Lung fibroblasts were cultured on different stiffness of polyacrylamide hydrogels for 72 h. The numbers of cells per 10 fields were manually counted. Values are means ± SD of six independent experiments. *P <* *0.05 vs. 1 kPa indicates significantly different.

Cell numbers were manually counted 72 h after they were seeded on different stiffnesses of polyacrylamide gels. Fibroblasts did not proliferate on 1 kPa gels (Fig. 4). Numbers of cells cultured on 2, 8, 25, and 50 kPa gels were significantly higher than those on 1 kPa gels. There was no significant difference in cell numbers between 2 and 50 kPa gels.

### Effects of substrate stiffness on expression of α‐smooth muscle actin

**Figure 5:**
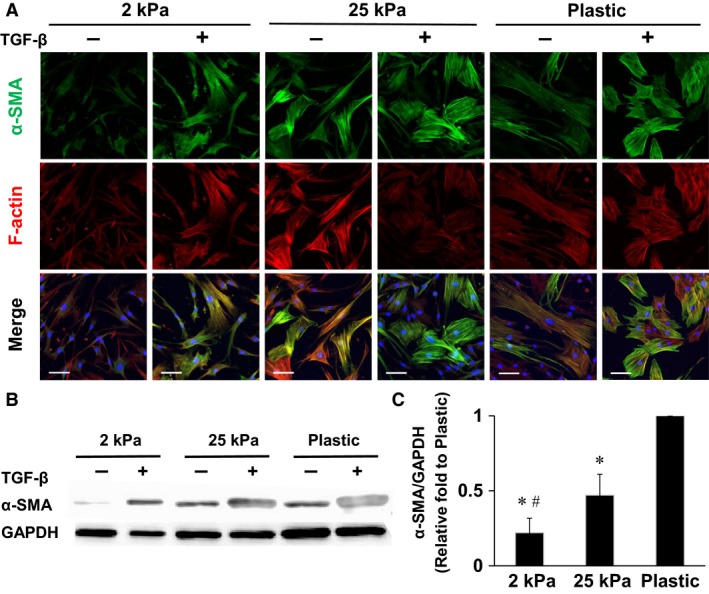
Substrate stiffness regulates expression of *α*‐SMA and F‐actin. (A) Representative immunofluorescence images of lung fibroblasts cultured on increasing substrate stiffnesses with or without TGF‐*β*_1_ (10 ng/mL) for 4 days, stained for *α*‐SMA (green), F‐actin (red), and nuclei (blue). Images were obtained using a confocal microscopy with a 25× objective. (B) Effects of substrate stiffness and TGF‐*β*_1_ (10 ng/mL) on expression of *α*‐SMA proteins as assessed by Western blotting. (C) *α*‐SMA protein/GAPDH protein ratios on different substrates without TGF‐*β*_1_ treatment were compared (*n* = 5). The *α*‐SMA/GAPDH ratio of the cells cultured on plastic dishes was defined as 1. Values are means ± SD. *P <* *0.05 vs. plastic dish and #*P *<* *0.05 vs. 25 kPa indicate significantly different. Bar = 100 *μ*m.

We investigated whether matrix stiffness induces differentiation of fibroblasts to myofibroblasts. We used 2 and 25 kPa gels as substrates of normal and fibrotic human lung tissues according to findings in previous literatures (Booth et al. 2012; Hinz 2012). Expression of *α*‐SMA protein was used as an indicator of myofibroblasts. The cells stimulated by 10 ng/mL TGF‐*β*_1_ (100‐21C; PeproTech, Rocky Hill, NJ) were used as a positive control for *α*‐SMA expression. Figure 5A shows immunofluorescent images of *α*‐SMA‐positive stress fibers, fluorescent F‐actin, and merged images of fibroblasts cultured on 2 and 25 kPa gels and plastic dish for 4 days in the presence or absence of 10 ng/mL TGF‐*β*_1_ (Fig. 5A). F‐actin stress fibers but not *α*‐SMA‐positive ones were observed in fibroblasts cultured on 2 kPa gels without TGF‐*β*_1_ (Fig. 5A). In contrast, treatment of the cells on 2 kPa gels with TGF‐*β*_1_ induced expression of *α*‐SMA‐positive stress fibers (Fig. 5A). Similar to the results of immunofluorescent images, there was little expression of *α*‐SMA protein as assessed by Western blotting in the cells on 2 kPa gels without TGF‐*β*_1_ (Fig. 5B). Levels of *α*‐SMA protein/GAPDH ratio in the cells cultured on 25 kPa gels and plastic dish without TGF‐*β*_1_ for 4 days were significantly more than those on 2 kPa gels (Fig. 5C).

### Effects of substrate stiffness on cell migration

**Figure 6:**
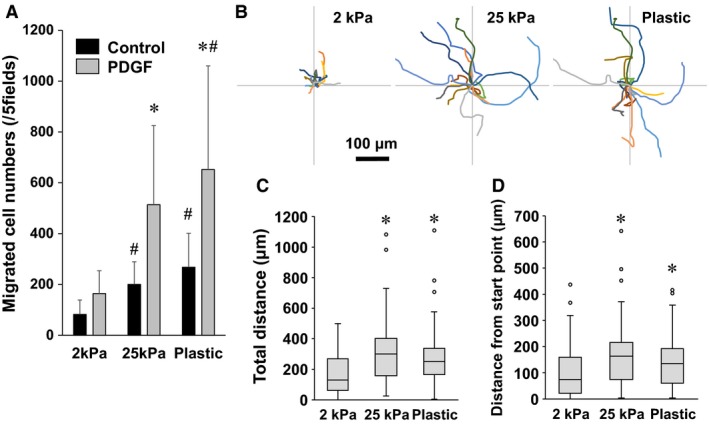
Effect of substrate stiffness on migration. (A) Lung fibroblasts precultured on different substrate (2 kPa and 25 kPa gels and plastic dishes) for 4 days were transferred to the wells of the Chemotaxicell chamber. The cells were stimulated by PDGF‐BB (10 ng/mL) or vehicle (control) for 6 h, and migrated cell numbers in five fields were counted. Values are means ± SD (*n* = 7). *P <* *0.05 vs. the control condition without PDGF‐BB and ^#^ *P *<* *0.05 vs. 2 kPa in each condition indicate significantly different. (B) Wind rose plots of random walk migration assays show centroid tracks of 15 representative cells from each indicated condition, with the initial position of each track superimposed on a common origin. Fibroblasts precultured on different substrates for 4 days were transferred to plastic dishes. One hour after seeding, phase‐contrast images were obtained every 20 min for a total of 12 h per experiment. Comparison of quantitative migration characteristics, (C) total migration distance and (D) distance from the start point. Boxes represent the 25th and 75th percentiles; whiskers indicate 10th and 90th percentiles of 90 cells for each indicated condition from four independent experiments. *P <* *0.05 vs. 2 kPa indicates significantly different.

The effects of substrates stiffness on migration of fibroblasts were examined by a transwell chemotaxis assay. Fibroblasts were cultured on polyacrylamide gels (2 or 25 kPa) or plastic dishes for 4 days before the assay. PDGF‐BB (10 ng/ mL) treatment significantly enhanced cell migration compared with time‐matched control cells without PDGF‐BB on 25 kPa gels and plastic dishes (Fig. 6A). The stimulation by PDGF‐BB significantly increased the numbers of cells in a stiffness‐dependent manner (Fig. 6A). The numbers of migrated cells on 25 kPa gels and plastic dishes without PDGF‐BB treatment were significantly higher than those on 2 kPa gels (Fig. 6A).

Next, we investigated the effects of substrate stiffness on random walk cell migration. Fibroblasts that had been cultured on different substrates for 4 days were seeded onto plastic dishes for the assay. Representative traces of migrating cells are shown in Figure 6B. Total migration distance and distance from the start point of the cells cultured on 25 kPa gels and plastic dishes were significantly more than those of the cells cultured on 2 kPa gels (Fig. 6C and D).

### Role of α‐smooth muscle actin in cell migration

**Figure 7:**
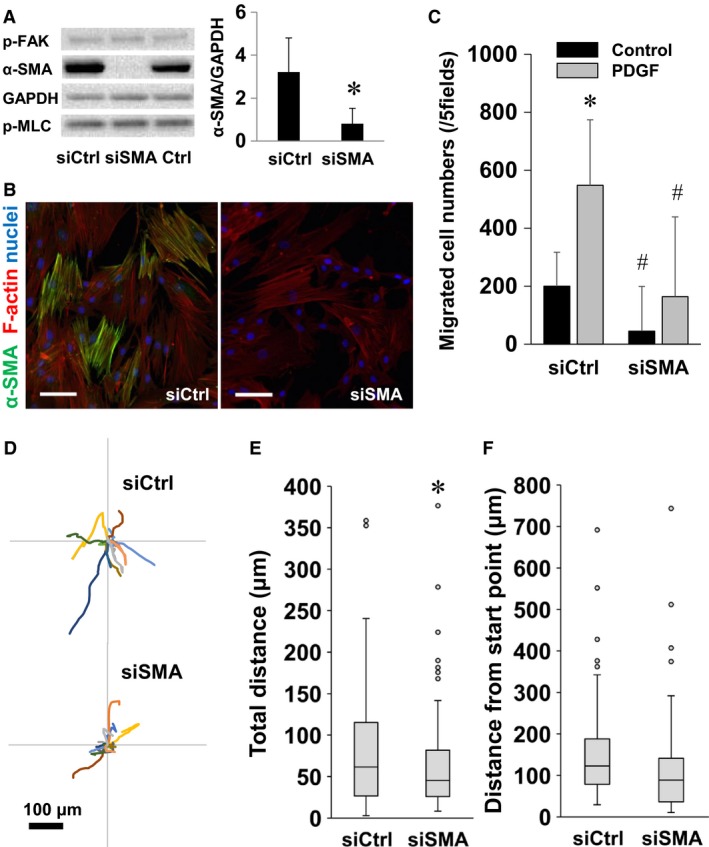
Roles of *α*‐SMA in regulation of fibroblast migration. (A) Western blotting of *α*‐SMA, phosphorylated (p)‐FAK (Tyr397), phospho‐myosin light chain (Ser19), and GAPDH to evaluate the knockdown efficiency of scrambled siRNA (siCtrl) and *α*‐SMA siRNA (siSMA) on plastic dishes. Values are means ± SD (*n* = 3). *P < 0.05 vs. siCtrl indicates significantly different. (B) Immunofluorescence images stained for *α*‐SMA (green), F‐actin (red), and nuclei (blue). Images were obtained using a confocal microscopy with a 25× objective. Bar = 100 *μ*m. (C) Fibroblasts precultured on plastic dishes and transfected with siSMA or siCtrl were transferred to the wells of a Chemotaxicell chamber. The cells were stimulated by PDGF‐BB (10 ng/mL) or vehicle for 6 h, and migrated cell numbers of five fields were counted. Values are means ± SD (*n* = 5). *P <* *0.05 vs. control without PDGF‐BB and #*P *<* *0.05 vs. siCtrl in each condition indicate significantly different. (D) Wind rose plots of random walk migration assay show centroid tracks of 15 representative cells from each indicated condition, with the initial position of each track superimposed on a common origin. Fibroblasts cultured on plastic dishes and transfected siSMA or siCtrl were transferred to plastic dishes. One hour after seeding, phase‐contrast images were obtained every 20 min for a total of 12 h per experiment. Comparison of quantitative migration characteristics, (E) total migration distance and (F) distance from the start point. Boxes represent the 25th and 75th percentiles; whiskers indicate 10th and 90th percentiles of 75 cells for each indicated condition from three independent experiments. *P <* *0.05 vs. siCtrl indicates significantly different.

We examined whether *α*‐SMA is involved in the mechanisms of migration of lung fibroblasts. Fibroblasts seeded on plastic dishes were transfected with siRNA targeting *α*‐SMA (siSMA) or scrambled siRNA. Levels of *α*‐SMA protein assessed by Western blotting 72 h after the transfection were significantly lower in the cells transfected with siSMA than the control cells transfected with scrambled siRNA (Fig. 7A). In contrast, levels of phosphorylated FAK (p‐FAK) and phosphorylated MLC (p‐MLC) were not affected by siSMA transfection (Fig. 7A). Immunofluorescent images show that expression of *α*‐SMA was inhibited, but that F‐actin formation was preserved in the fibroblasts transfected with siSMA (Fig. 7B). Results of the transwell chemotaxis assay showed that migrated cell numbers treated with and without PDGF‐BB were significantly fewer in fibroblasts transfected with siSMA than those transfected with scrambled siRNA (Fig. 7C). Representative traces of random walk migration of fibroblasts transfected with siSMA or scrambled siRNA are shown in Figure 7D. Results of random walk migration assays show that total migration distances of the cells transfected with siSMA were significantly less than those of the control cells (Fig. 7E).

## Discussion

The main findings of the present study are that (1) as substrate stiffness increases, lung fibroblasts spread with the formation of stress fibers positive for *α*‐SMA; (2) protein expression of *α*‐SMA was significantly higher on stiff substrates than that on soft substrates; (3) cell migration assessed by chemotaxis and random walk assays was significantly enhanced by stiff substrates; and (4) transfection with siRNA for *α*‐SMA inhibited PDGF‐BB‐induced chemotaxis and random walk cell migration. To our knowledge, we demonstrated for the first time that a stiff matrix induces high motility with *α*‐SMA expression and that *α*‐SMA is involved in the mechanisms of cell migration in human lung fibroblasts.

In the morphological study, cell projection area, aspect ratio, and perimeter were increased and circularity was decreased in stiffness‐dependent manners, which resulted in increased numbers of spread and spindle‐shaped fibroblasts on stiff substrates (Figs. 1 and 2). The expression of *α*‐SMA protein increased with substrate stiffness (Fig. 5). Cell spreading is tightly coupled to proliferation in lung fibroblasts (Mih et al. 2012). It has been demonstrated that a stiff substrate induces proliferation, cell spreading, traction generation, and differentiation to myofibroblasts in lung fibroblasts (Huang et al. 2012; Mih et al. 2012; Marinkovic et al. 2013). *α*‐SMA is one of the six known eukaryotic actin isoforms that is found predominantly in smooth muscle (Vandekerckhove and Weber 1978), but also expressed in other specialized cells such as pericytes and myofibroblasts (Skalli et al. 1986; Rockey et al. 2013). Acquisition of *α*‐SMA expression characterizes fibroblast‐to‐myofibroblast differentiation and increased contractile ability of fibroblasts (Kuhn and McDonald 1991; Hinz 2001; Hinz et al. 2012). Consistent with findings in previous reports (Liu et al. 2010; Balestrini et al. 2012; Huang et al. 2012; Zhou et al. 2013), we demonstrated that the morphology, proliferation, and differentiation of lung fibroblasts are regulated by rigidity of substrates.

Cell migration as assessed by chemotaxis and random walk migration was significantly enhanced when fibroblasts were cultured on stiff substrates (Fig. 6). Cell proliferation was also affected by substrate stiffness (Fig. 4). One of the histological characteristics of pulmonary fibrosis is accumulation of lung fibroblasts and myofibroblasts (King et al. 2011). Various factors such as proliferation of resident fibroblasts, differentiation of fibroblasts to myofibroblasts, and migration of lung fibroblasts and myofibroblasts from the normal lesion to the fibrotic lesion are involved in the mechanisms of the accumulation of fibroblasts and myofibroblasts (Tschumperlin 2013; Barkauskas and Noble 2014). Tsukui et al. (2013) reported that migration of lung fibroblasts is more important than their proliferation in a murine model of bleomycin‐induced lung injury. Previous in vitro studies have demonstrated that lung fibroblasts isolated from patients with IPF show higher motility than those from normal subjects (Suganuma et al. 1995; White et al. 2003b). These findings together with our results suggest that lung fibroblasts acquire higher migrating ability under stiff substrate conditions. Lo et al. (2000) first demonstrated that NIH3T3 fibroblasts migrate preferentially toward a rigid substrate using an in vitro 2D model. This preference for a stiff substrate in cell migration is called “durotaxis” (Lo et al. 2000; Kobayashi and Sokabe 2010). It is considered that lung fibroblasts migrate toward and accumulate in fibrotic lesions in pulmonary fibrosis where they differentiate into myofibroblasts, possibly via durotaxis (Tsukui et al. 2013; Southern et al. 2016). Thus, as the motile properties of lung fibroblasts, durotaxis might also be involved in the mechanisms of pathophysiology of pulmonary fibrosis.

As we postulated, decreases in *α*‐SMA proteins by siRNA transfection inhibited migration (Fig. 7). Rockey et al. (2013) reported that upregulation of *α*‐SMA is associated with enhanced motility in hepatic myofibroblasts isolated from a damaged liver and that hepatic fibroblasts isolated from *α*‐SMA‐null mice exhibit reduced motility. Fibroblast migration in 2D substrates is characterized by a multistep cycle of protrusion, adhesion formation, and stabilization at the leading edge, followed by cell body translocation and release of adhesions and detachment of the rear of the cell (Tschumperlin 2013). It is established that a focal adhesion complex, Rac1, RhoA/Rho‐kinase, and cell contraction are related to fibroblast migration (Huttenlocher and Horwitz 2011; Bordeleau and Reinhart‐King 2016; Chen et al. 2016a). In addition, myosin II and phosphorylation of MLC are also key regulators of migration of lung fibroblasts (Southern et al. 2016). In our results, neither phosphorylation of FAK nor MLC was inhibited by transfection of siRNA for *α*‐SMA (Fig. 7). Moreover, actin stress fiber formation was also preserved in the fibroblasts transfected with siSMA (Fig. 7B). Our findings demonstrated that *α*‐SMA plays a pivotal role in mechanisms underlying migration of lung fibroblasts. In contrast to our findings, expression of *α*‐SMA is related to a decrease in motility of human breast fibroblasts (Ronnov‐Jessen and Petersen 1996). Chen et al. (2016b) reported that overexpression of *α*‐SMA expression inhibits migration of vascular smooth muscle cells from mice. Southern et al. (2016) utilized their unique murine lung tissue model to explore the effects of normal (soft) and fibrotic (stiff) lung matrix on *α*‐SMA expression and random walk migration of normal lung fibroblasts. They demonstrated that *α*‐SMA expression is upregulated, but fibroblasts are immobilized on the fibrotic lung tissue isolated from a murine model of bleomycin‐induced lung injury. Thus, the contribution of *α*‐SMA to motility may depend on cell type, experimental condition, material for substrate, and fibroblast heterogeneity (Phan 2008). We also note that migration was not completely inhibited in fibroblasts which expressed no or few *α*‐SMA proteins (Figs. 6 and 7). These results suggest that *α*‐SMA‐independent pathways are also involved in mechanisms underlying matrix stiffness‐induced migration of lung fibroblasts. Therefore, future studies are important to sort out the mechanisms including relative importance of *α*‐SMA and matrix stiffness in defining the migratory phenotype.

There are multiple mechanisms underlying expression of *α*‐SMA (Hinz et al. 2012). In addition to chemical stimuli, specifically TGF‐*β*, mechanical cues such as traction force and contraction of the fibroblasts are important for *α*‐SMA expression (Hinz 2012). Incorporation of *α*‐SMA into stress fibers results in upregulation of contractile activity (Hinz 2001). Taken together, it is likely that positive feedback via activating cellular mechanotransduction (Liu et al. 2010; Parker et al. 2014) is involved in the mechanisms of differentiation, *α*‐SMA expression, and increased migration induced by matrix stiffness in lung fibroblasts.

In the present experimental protocols, fibroblasts were precultured on substrates of different stiffness for 3 days and then seeded onto stiff conditions, either a plastic dish or chemotaxis chamber, for migration assays. It has been reported that fibroblasts primed on various levels of stiffness possess a “mechanical memory,” at least for a few days (Balestrini et al. 2012). In our experiments, fibroblasts precultured on a stiff matrix exhibited faster motility in both chemotaxis and random walk assays than those on soft gels, indicating that the mechanical memory due to mechanical priming on the stiff condition contributes to migrating properties of lung fibroblasts. It would also be important to examine migrating abilities on the gel without transferring the cells to different environments. However, it is difficult to directly compare migrating ability of the cells on matrix of different stiffness. Future studies are necessary.

In summary, morphology, proliferation, expression of *α*‐SMA, and motility of human lung fibroblasts were affected by matrix stiffness. Additionally, inhibition of *α*‐SMA expression with siRNA reduced migration properties. Activation of lung fibroblasts by the stiff lung and positive feedback may be involved in the mechanisms of progression of pulmonary fibrosis.

## Conflict of Interest

None declared.
